# Analysis of unscheduled remote monitoring transmissions from patients with cardiac implantable electronic devices attending a heart failure service

**DOI:** 10.1093/europace/euae297

**Published:** 2024-12-16

**Authors:** Nick S R Lan, Alicia Donovan, James Lambert, Lawrence Dembo, Amit Shah, Vimal Patel

**Affiliations:** Department of Advanced Heart Failure and Cardiac Transplantation, Fiona Stanley Hospital, 11 Robin Warren Drive, Murdoch, Perth, Western Australia 6155, Australia; Medical School, The University of Western Australia, Perth, Western Australia, Australia; Department of Advanced Heart Failure and Cardiac Transplantation, Fiona Stanley Hospital, 11 Robin Warren Drive, Murdoch, Perth, Western Australia 6155, Australia; Department of Advanced Heart Failure and Cardiac Transplantation, Fiona Stanley Hospital, 11 Robin Warren Drive, Murdoch, Perth, Western Australia 6155, Australia; Department of Advanced Heart Failure and Cardiac Transplantation, Fiona Stanley Hospital, 11 Robin Warren Drive, Murdoch, Perth, Western Australia 6155, Australia; Department of Advanced Heart Failure and Cardiac Transplantation, Fiona Stanley Hospital, 11 Robin Warren Drive, Murdoch, Perth, Western Australia 6155, Australia; Department of Advanced Heart Failure and Cardiac Transplantation, Fiona Stanley Hospital, 11 Robin Warren Drive, Murdoch, Perth, Western Australia 6155, Australia; School of Human Science, The University of Western Australia, 35 Stirling Highway, Crawley, Perth, Western Australia 6009, Australia

**Keywords:** Cardiac devices, Remote monitoring, Transmissions, Heart failure

**What’s new?**

- Unscheduled remote transmissions generated a large workload but resulted in few management changes in patients attending a devices clinic within an advanced heart failure service.

Remote monitoring of cardiac implantable electronic devices (CIEDs) allows timely recognition of patient and device events and is guideline recommended.^1^ A recent international consensus statement gives a Class 2A recommendation for remote monitoring to be conducted in patients with heart failure to monitor heart failure diagnostics and detect progression of heart failure.^2^ Remote monitoring requires appropriate staffing and adequate resources. The increasing number of patients with CIEDs and volume of transmissions creates significant workload for device clinics as each transmission requires timely attention and can result in patient and clinician communication, clinical action, and documentation.^3^ Thus, further research to determine optimal models of remote monitoring clinics have been advocated.^2^ Recent studies have shown that the burden of remote transmissions in the general cardiology patient group is high, yet in one study, only 7% of alerts were judged to be clinically meaningful.^4,5^ The proportion of complex devices in patients attending heart failure clinics are higher, possibly resulting in more device alerts and subsequent management changes. We therefore sought to describe the real-world burden of unscheduled transmissions from CIEDs and management changes in patients attending a heart failure service.

This was a retrospective single-centre study of remote transmissions during the year 2023 from patients with CIEDs attending a dedicated devices clinic within the Western Australian Advanced Heart Failure and Cardiac Transplant Service. At our institution, remote follow-up of CIEDs and implantable loop recorders is implemented in accordance with recommendations.^2^ All transmissions were evaluated by a device specialist certified by the International Board of Heart Rhythm Examiners. Management changes (either programming or medication changes) were made in conjunction with treating cardiologists, including electrophysiologists where required. If management changes were required, the patient was scheduled for an in-person review or managed through phone consultations depending on the type of alert received and the clinical context. We calculated the costs of supporting a CIED clinic based on recommendations of 3.0 full-time equivalent for 1000 CIED patients and an estimated average annual Australian wage of $123 000AUD ($81 546.00USD) for Cardiac Physiologists.^2^ Data are descriptive and the study was approved by the local institution (FSFHG GEKO52414).

Of 5187 transmissions from 315 patients [mean age 56.4 ± 13.2 years and 226 (71.7%) males], 2723 (52.5%) were unscheduled from 255 (81.0%) patients (*Figure 1*). Of these 255 patients, 9 (3.5%) had a permanent pacemaker, 132 (51.8%) had an implantable cardioverter defibrillator (ICD), 100 (39.2%) and 7 (2.7%) had a cardiac resynchronization therapy (CRT) with ICD or pacemaker respectively, and 7 (2.7%) had a loop recorder. Unscheduled transmissions included ventricular tachycardia/fibrillation (*n* = 28; 1.0%), non-sustained ventricular tachycardia (*n* = 355; 13.0%), atrial fibrillation or supraventricular tachycardia (*n* = 868; 31.9%), lead issues (*n* = 454; 16.7%), sub-optimal CRT pacing (*n* = 129; 4.7%), heart failure alerts (*n* = 241; 8.9%), patient-initiated (*n* = 468; 17.2%), and others (*n* = 180; 6.6%). Unscheduled transmissions led to 54 (2.0%) patient encounters, resulting in 2 (0.1%) hospital admissions, 16 (0.6%) programming changes, and 26 (1.0%) medication changes. Medication changes were most commonly (69.2%) made due to detection of ventricular tachycardia/fibrillation. The estimated cost of staffing our CIED clinic was $116 235.50AUD ($77 061.45USD) for 0.945 full-time equivalent wage for a Cardiac Physiologist.

**Figure 1:**
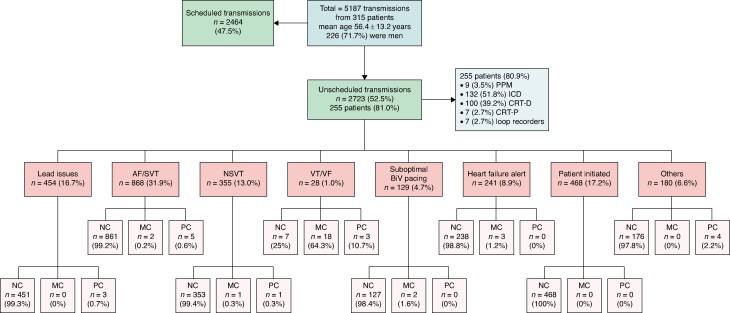
Summary of findings from unscheduled remote transmissions and resulting management changes. AF, atrial fibrillation; BiV, biventricular; CRT-D, cardiac resynchronization therapy with defibrillator; CRT-P, cardiac resynchronization therapy with pacemaker; ICD, implantable cardioverter defibrillator; NC, no change; NSVT, non-sustained ventricular tachycardia; MC, medication change; PC, programming change; PPM, permanent pacemaker; SVT, supraventricular tachycardia; VF, ventricular fibrillation; VT, ventricular tachycardia.

There is increasing interest in the use of remote monitoring in patients with heart failure to predict decompensation and therefore trigger earlier action.^6,7^ Our study describes the remote monitoring workload of a dedicated device clinic within a heart failure service, where ∼90% of patients had an ICD or CRT with ICD. Importantly, studies have shown that remote monitoring in patients with ICD or CRT with ICD is associated with a reduction in cardiac and all-cause mortality compared with traditional office visits.^8^ In our cohort, unscheduled remote transmissions generated a large workload for staff, but resulted in management changes in less than 2% of patients. Heart failure alerts led to few management changes, which could be due to sub-optimal specificity of these algorithms in isolation to detect decompensation.^3,9^ This highlights the importance of integrating remote monitoring data with the medical history and other investigations when considering whether management changes are required.^6^ Nonetheless, remote monitoring in our cohort allowed for timely detection and management of ventricular arrhythmias.

Our findings underscore the need for personalization of alert triggers, such that potentially unnecessary remote transmissions or non-actionable alerts can be reduced. However, the variability between CIED manufacturers on programmable alerts may be a barrier to developing standardized workflow models. Multiparametric algorithms that combine several CIED parameters may help to predict worsening heart failure, but ongoing research is required to determine how they are managed, their impact on clinical outcomes and costs.^6,7^ Rapid advancements in digital technologies have led to the development of automated software, artificial intelligence and third-party data management systems which could be used to reduce staff workload in device clinics by triaging transmissions and identifying alerts requiring clinical intervention.^2,10^ These solutions could be leveraged to improve the cost-effectiveness of remote monitoring, as a lack of reimbursement poses a significant barrier to the widespread implementation of remote monitoring in many countries.^7^ Here, our data show that significant costs are incurred to review unscheduled transmissions despite the low proportion requiring management changes.^7^

Limitations of our study include the retrospective analysis and single-centre cohort. We did not evaluate whether unscheduled transmissions led to additional laboratory or diagnostic tests. The cost of our service may be underestimated, as it does not account for the additional resources required to attend to each patient encounter and subsequent management changes. The low number of interventions observed may be because this cohort of patients were receiving optimal treatment at the specialized heart failure service. As such, management changes due to unscheduled transmissions were often not required. Data from our study may not be applicable to other centres with differences in care delivery. Lastly, we did not evaluate the impact of remote transmissions that did not result in management changes; such transmissions may have provided patient reassurance and reduced anxiety levels whilst also preventing urgent care visits and subsequent healthcare costs.

In conclusion, this contemporary real-world study shows that unscheduled remote transmissions generate a large workload, but resulted in few management changes, in patients attending a dedicated devices clinics within an advanced heart failure service. Further research should address the high burden of non-actionable unscheduled transmissions, thereby addressing the growing challenge of resource allocation and structured device clinic workflow models in this patient population.

## Data Availability

The data sets generated and/or analysed during the current study are not publicly available to maintain patient confidentiality but are available from the corresponding author on reasonable request and after the agreement of all the co-authors.
